# Can the Relationship between Doctors and Drug Companies Ever Be a Healthy One?

**DOI:** 10.1371/journal.pmed.1000075

**Published:** 2009-07-21

**Authors:** Emma D'Arcy, Ray Moynihan

**Affiliations:** 1 Scientific Insights, myPHID Limited, Kent, United Kingdom; 2 School of Medicine and Public Health, University of Newcastle, Newcastle, New South Wales, Australia

## Abstract

Emma D'Arcy and Ray Moynihan debate whether doctors and drug companies can form healthy alliances or whether these will always be prone to the corrupting influence of drug company money.

## Background to the debate

The financial ties between doctors and drug companies have come under intense scrutiny in recent years. Some commentators—such as Marcia Angell, former editor-in-chief of the *New England Journal of Medicine*—argue that the mission of doctors is fundamentally different to the mission of drug companies and that the ties between them should be completely cut. “Drug companies are investor owned businesses with a responsibility to maximise profits for their shareholders,” says Angell [1]. “That is quite different from the mission of the medical profession, which is to provide the best care possible for patients.” Other commentators have argued that clinicians and drug companies do have some shared goals in aiming to maximize human health. In this debate, Emma D'Arcy, co-founder of a social networking site that facilitates interactions between doctors and drug companies, argues that it would be valuable to the public if we could establish “authentic alliances” between these professionals. But journalist Ray Moynihan argues that such alliances are prone to the corrupting influence of pharmaceutical industry money, and that disentanglement is a healthier alternative.

## Emma D'Arcy's Viewpoint: We Should Embrace a New Era of Engagement and Shared Aspirations

The relationship between doctors and drug companies is the subject of intense scrutiny—there is widespread skepticism about the intent of industry and concern for the vulnerability of doctors in the relationship. Unfortunately, the debate on how to move this relationship forward has become polarized: industry argues that collaboration with physicians is essential to scientific advancement, but at the same time many doctors are pledging to cut all their ties to drug companies (see, for example, the No Free Lunch pledge at http://www.nofreelunch.org/pledge.htm).

But there is surely one thing we can all agree upon: both the pharmaceutical industry and health care professionals must focus on the goal of improving health. We seem to have lost sight of the *shared* aspirations between medical and pharmaceutical professionals. It is in everyone's interest that medicines are safe and effective. It is not credible to imply that health care professionals are easy victims to an industry that readily fools them with its marketing tactics. Many physician leaders find it condescending to be considered so malleable to “marketing exercises,” and it is offensive to suggest they cannot conduct an ethical exchange with industry. Despite strong differences between doctors and drug companies, now is surely a critical time to determine how to establish and nourish authentic alliances between these professionals.

The media prefer to publish stories about unsafe drugs, doctors who succumb to financial incentives, and sensationalized accounts of drug companies suppressing unfavorable trial data. These news stories fuel public fear that interactions with industry are eroding physicians' professionalism. Yet there are many examples where industry has acted purely in the public interest with no expectation of financial rewards. For example, many drug companies readily award grants for scientific meetings that are important to the research community without expectation that promotional information will be included. In addition, the pharmaceutical industry is an extremely important source of funding for continuing medical education—35% of the estimated US$9–US$14 billion that industry spends each year on pharmaceutical marketing goes towards educational support [2]. If pharma-sponsored education is no longer allowed, we may witness tomorrow's doctors practicing yesterday's medicine.

It is in industry's best interests to develop drugs that help health care professionals excel in treating their patients. Each drug company must achieve such drug development within a highly regulated environment where governments, trade associations, professional societies, and individual company codes of practice apply to protect scientific integrity. Medical innovation may be hindered if we further limit interactions. Medical professionals and industry researchers may find it equally frustrating if restrictions on interactions limit their professional aspirations.

The pharmaceutical industry is held responsible for the rising costs of health care. But surely industry deserves to be rewarded for the financial risks they have taken to develop new prescription drugs. It is easy to forget that products developed by industry have consistently improved human health for three decades. Industry capitalizes on our desire to live longer, healthier lives; it is arguably a victim of its own success in meeting our desires. When industry and physicians collaborate, the most likely result is expediency in producing new treatments. The first step to recover the value of such collaboration is to accept that *both* parties must assume accountability for the transparency and outcomes of their interactions.

I believe that there are three ways in which we can build healthier relationships between doctors and drug companies without having to introduce yet more stringent regulations.

### Teaching Physicians “Promotional Literacy”

Medical professionals should be taught to distinguish between sound clinical information and promotional materials. They should be able to assess, for example, the quality of a clinical trial and whether the outcome could have been unduly influenced by commercial forces.

### Encouraging the Adoption of “Good Relationship Practice”

Health and pharmaceutical professionals should both follow a set of rules to guide healthy interactions. This could take the form of three simple questions—an “everyday credo” to ask oneself before attempting to engage in a new physician–pharma alliance (see Box 1).

**Box 1. Three Questions That Should Be Asked Before Engaging in a Physician–Pharma Alliance**

- Does this interaction, or series of interactions, encourage scientific exchange of information or lead to an enhanced skill that will ultimately benefit the care of people living with disease and/or enhance the knowledge of those aspiring to help people to overcome, manage, or better understand a medical condition?
- Does this interaction require the individuals to have a good knowledge of the drug development process and/or an understanding about how industry uses promotional messaging of clinical data?
- Is there any possibility that this interaction could be viewed as an inappropriate activity that could damage the perception of any of the participants' intentions and integrity to engage in a positive collaboration that furthers medical scientific understanding?

### Ensuring Transparency from Both Parties, While Allowing Healthy Networking

Both parties must be transparent about combining research, clinical, and educational endeavors within a framework of professional networking. Such networking could be assisted by online professional networking facilities such as http://www.myphid.com/ and http://www.medcrowd.com/.

Responsible leadership is no longer about influencing opinion but is instead about aligning aspirations and realizing ambitions. Doctors champion patient needs, and pharma requires a positive presence in health care committed to addressing these needs. Both groups want to make necessary moves to improve interactions. Embracing a new era of engagement and acknowledging the positive aspects of aligning will move this debate from rhetoric to reality.

## Ray Moynihan's Viewpoint: Disentanglement Will Deliver a Healthier Relationship between Doctors and Drug Companies

The idea of establishing and nourishing “authentic alliances” between doctors and drug companies, in order to embrace a “new era of engagement,” sounds extremely enticing. Surely, as Emma D'Arcy eloquently argues, it is time to put behind us all this tiresome talk of physicians being in bed with pharmaceutical companies, and focus instead on the “shared aspirations” to improve human health. Sadly, her Viewpoint is more beguiling than revealing, drawing on the marketing catchphrases of her Web site (http://www.myphid.com/) rather than addressing the crude financial question at the heart of this debate: who pays for the pizza?

Healthy new relationships can only be built by a medical profession that disentangles itself from the corrupting influence of the billions it accepts annually from the marketing budgets of big pharmaceutical companies. Whether it's the free pizza at grand rounds, the inflated honoraria that “key opinion leaders” take for promoting the latest pills, or the prestigious contracts for sponsored “scientific research” often driven more by shareholder interest rather than public need, ending such entanglement is surely one way to deliver a healthier relationship between doctors and drug companies.

Certainly given the high profile of Senator Charles Grassley's investigations (he recently urged the American Psychiatric Association to disclose its drug industry ties [3]), and Pfizer's recent announcement that it was considering disclosing payments of at least $US500/year to doctors [4], you could be forgiven for thinking we are living in a new era of transparency. A few short years ago such basic transparency mechanisms would have been seen as unworkable and unnecessary. But disclosing ties that can distort prescribing behavior, such as those shown in a study by Ashley Wazana [5], is not the same as disentangling them.

I worry that Emma D'Arcy's Viewpoint may in fact be part of a new era of public relations rhetoric designed to persuade physicians and policy makers that there *is* such a thing as a free lunch, and that cutting ties with drug companies is a risky business. I suspect too that we are likely to hear a lot more noise about how doctors and their associations can continue to accept pharmaceutical industry money without it perverting their professional practice. And a lot of doctors will be receptive to that rhetoric because there is a lot more at stake here than giving up access to free pens. Drug company largesse still lubricates the lifestyles of many physicians, funding the fancy meals and the five-star hotel rooms, sponsoring the medical education and the scientific conferences, facilitating the prestigious research and the all-important publications.

A key obstacle to cleaning up this mess is that many of the so-called “key opinion leaders” who “educate” their peers at seminars and conferences are living a lie: pretending they can simultaneously serve the private interests of their sponsors and the public interest that is supposed to flow from their academic and professional responsibilities. As the recent Josiah Macy, Jr. Foundation report observed, the responsibilities of doctors and drug companies are “fundamentally incompatible” [6] and there should be a comprehensive ban on all industry funding of continuing medical education.

Doctors gaining mandated professional credits by listening to company-sponsored speakers at company-sponsored events organized by company-sponsored associations is an obscene perversion of education that is an embarrassment to all involved. To claim with a straight face that drug and device makers sponsor such “education” as some kind of selfless service, or that medical practice will suffer if doctors are educated in independently funded forums, is as bizarre as it is unbelievable.

Similarly, just as doctors are starting to seek out educational activities genuinely free of drug company funding and influence, so too, more prescribers are starting to politely decline the opportunity to be fed and “informed” by drug company salespeople. For several years now the American Medical Student Association's “PharmFree” strategy has advocated the severing of many of these financial ties, prefiguring a future where far fewer doctors will be prescribing under the influence of industry [7].

In my view the nascent moves towards disentanglement witnessed in recent years—in hospitals, universities, and professional associations—will only intensify, as new disclosure regimes attract even more public attention to these unhealthy relationships. Visits by “sexy” sales representatives, drug-sponsored continuing medical education, or scientific conferences in exotic locations could very quickly become laughable images of a bygone era.

The question of how to build relationships around genuine research is more complex. As the *BMJ* editor Fiona Godlee said in a recent special issue devoted to this topic, doctors should engage only in research and clinical collaborations that are “transparent and unbiased in their design and reporting” [8]. But they must also say no, she argued, to all gifts and hospitality, and should refuse to be ghost authors, decline any roles as paid opinion leaders, and fund their own education and information.

One of the basic rules of journalism is follow the money. The problem with Emma D'Arcy's Viewpoint is its lack of clarity on the appropriateness of the myriad flows of money between doctors and drug companies. Similarly, despite the “transparency” rhetoric, it is not immediately clear who is paying the bills for her own social networking Web site, which states: “welcome to a new era and environment that recognises and rewards scientific collaborations of merit.” A detailed list of sponsors and descriptions of what the contracts with those sponsors involve would be very welcome.

## Emma D'Arcy's Response to Ray Moynihan's Viewpoint

While it is a pleasure to be described by Ray Moynihan as eloquent, it is a shame that this debate defaults to cynical sniping. I am not a secret PR strategist for industry. Nor am I enjoying great commercial success in trying to create an environment where unproductive mud-slinging stops and all parties seek appropriate ways to work together.

Moynihan asks for a “detailed list of sponsors” of myPHID, and for Expert Sessions these are available online at www.myPHID.com. The aspirations of this Web site reflect what lies at the heart of this debate—behaving differently, behaving better. That's not a marketing slogan—it's common sense.

This new attitude requires all stakeholders to behave with respect as befits professionals. The pioneers from both the medical profession and the pharmaceutical industry featured on the Web site, for example, are highly respected and praised for their integrity. Doctors on myPHID often recommend members of the pharmaceutical industry, and vice versa, as beacons of scientific sincerity determined to improve patient outcomes. There is no “sexy” puppeteer manipulating their engagements—individual members of myPHID are simply choosing to work with others who excel in their chosen field. This cooperative dignity must be a fundamental working credo in seeking solutions to the challenges of interactions between the medical community and the pharmaceutical industry. Accordingly, I do not dictate the outputs of myPHID—the community that uses it to increase transparency and establish better alignments dictates them. Social networking methodologies lead to “user-generated” content and more equal conversations. Such conversations elevate the debate about physician–drug company interactions above the “victim–persecutor–rescuer” drama that has been given so much airtime and that is based on questionable evidence of any harm from such interactions. The “facts” cited by those who criticize physician–drug company relationships provide little substantiation that so-called “entanglements” are unhealthy. And thanks to “key opinion leaders,” industry-funded medical innovations reach patients.

As for Moynihan's call for a “comprehensive ban on all industry funding of continuing medical education,” this suggests that all industry professionals are devious and that all medical professionals are inducible. The issue at stake here is not whether industry funding of education is a selfless act—what matters is the *quality* of the educational content. If there truly is evidence that all company-sponsored education is, as Moynihan suggests, “an obscene perversion of education,” then let's see it. The myPHID community would want to see this evidence and use it to make changes.

Moynihan and I do agree on one thing—the lack of clarity in the relationships between doctors and drug companies needs to be addressed. But I believe that there will be no clear route forward if we simply continue to perpetuate the false idea that “working with pharma is bad.” Medical and pharmaceutical professionals alike should be applauded for committing time and energy to collaborative projects such as those that myPHID is trying to coordinate. If Ray Moynihan would like to sponsor an Expert Session on myPHID that truly opens this debate and allows articulation of all viewpoints, we would certainly be delighted to accept his sponsorship and make it accessible to all.

## Ray Moynihan's Response to Emma D'Arcy

Anyone genuinely interested in evidence about the dangers of entanglement need go no further than the Web site of the nonprofit group Healthy Skepticism (http://www.healthyskepticism.org/), which boasts a wealth of peer-reviewed data suggesting money does in fact buy influence. For example, one systematic review found that “studies sponsored by pharmaceutical companies were more likely to have outcomes favouring the sponsor than were studies with other sponsors,” pointing to a “systematic bias” in funded research [9].

Similarly, while “key opinion leaders” enthusiastically help medical innovations reach patients, the question is whether that enthusiasm always arises from dispassionate analysis of data, or whether it might be related to the promise of on-going lucrative consultancies. As long-time drug company insider Kimberley Elliott put it recently: “Key opinion leaders were salespeople for us, and we would routinely measure the return on our investment, by tracking prescriptions before and after their presentations. If that speaker didn't make the impact the company was looking for, then you wouldn't invite them back” [10].

Influence is obviously often far less crude than this, though a recent investigation suggests it is pervasive nonetheless, even affecting fully accredited educational events [11]. A popular provider of education for Australian doctors was for several years offering sponsors the explicit chance to help determine topics and speakers for seminars, sold to participants as “independent of industry influence” [11]. More importantly, industry representatives confirmed that it's not unusual for sponsors to suggest speakers for these educational events. Given this unhealthy influence of drug companies upon medical education, some journal editors believe the time has come for doctors to pay for their own education. Similarly, the authors of the 2008 Josiah Macy, Jr. Foundation report called for the “comprehensive ban” reported on in my Viewpoint above, suggesting that educational providers “should not accept any commercial support from pharmaceutical or medical device companies,” whether directly or indirectly [6].

Despite rumors to the contrary, doctors are human too, and as vulnerable as anyone else to the “food, flattery, and friendship” that comes with subsidized seminars, free trips, and fancy hotel rooms. Which is why getting out of bed with drug companies is not going to be easy. I know from bitter experience. After the Association of Health Care Journalists introduced its 2004 policy of not accepting for-profit health care company sponsorship [12], the quality of accommodation at its conferences seemed to decline somewhat. I remember arriving at a North Carolina conference, after a very long flight from Australia, and being forced to slum it in a single bed, in a shared room with a fellow writer who, unfortunately, had a bit of a snore. The conference, nevertheless, was a great success.

In 2004, the newly launched *PLoS Medicine* announced it would be free of drug company advertising, and would not profit from the business of exclusive reprint sales to the pharmaceutical industry. The editors adopted this position in order to ”break the cycle of dependency” between the medical profession and the pharmaceutical industry, and to create a “healthy” journal, where debates like this one take place in the sunshine of openness, not in the long shadows of industry influence [13]. This sort of disentanglement is one step towards a more healthy relationship between doctors and drug companies.
